# Vitamin D and Physical Performance in Elderly Subjects: The Pro.V.A Study

**DOI:** 10.1371/journal.pone.0034950

**Published:** 2012-04-23

**Authors:** Elena D. Toffanello, Egle Perissinotto, Giuseppe Sergi, Sabina Zambon, Estella Musacchio, Stefania Maggi, Alessandra Coin, Leonardo Sartori, Maria-Chiara Corti, Giovannella Baggio, Gaetano Crepaldi, Enzo Manzato

**Affiliations:** 1 Department of Medical and Surgical Sciences, Geriatrics Division, University of Padova, Padova, Italy; 2 Department of Environmental Medicine and Public Health, University of Padova, Padova, Italy; 3 Department of Medical and Surgical Sciences, University of Padova, Padova, Italy; 4 National Research Council, Aging Branch, Institute of Neuroscience, Padova, Italy; 5 Azienda Unità Locale Socio Sanitaria, Padova, Italy; 6 Division of Internal Medicine, Azienda Ospedaliera di Padova, Padova, Italy; University of Granada, Spain

## Abstract

**Background:**

The role of Vitamin D in musculoskeletal functionality among elderly people is still controversial. We investigated the association between serum 25-hydroxyvitamin D (25OHD) levels and physical performance in older adults.

**Methods:**

2694 community-dwelling elderly women and men from the *Progetto Veneto Anziani* (Pro.V.A.) were included. Physical performances were assessed by: tandem test, 5 timed chair stands (TCS), gait speed, 6-minute walking (6 mW) distance, handgrip strength, and quadriceps strength. For each test, separate general linear models and loess plots were obtained in both genders, in relation to serum 25OHD concentrations, controlling for several potential confounders.

**Results:**

Linear associations with 25OHD levels were observed for TCS, gait speed, 6 mW test and handgrip strength, but not for tandem test and quadriceps strength. After adjusting for potential confounders, linear associations with 25OHD levels were still evident for the 6 mW distance in both genders (p = .0002 in women; <.0001 in men), for TCS in women (p = .004) and for gait speed (p = .0006) and handgrip strength (p = .03) in men. In loess analyses, performance in TCS in women, in gait speed and handgrip strength in men and in 6 mW in both genders, improved with increasing levels of 25OHD, with most of the improvements occurring for 25OHD levels from 20 to 100 nmol/L.

**Conclusion:**

lower 25OHD levels are associated with a worse coordination and weaker strength (TCS) in women, a slower walking time and a lower upper limb strength in men, and a weaker aerobic capacity (6 mW) in both genders. For optimal physical performances, 25OHD concentrations of 100 nmol/L appear to be more advantageous in elderly men and women, and Vitamin D supplementation should be encouraged to maintain their 25OHD levels as high as this threshold.

## Introduction

Aging people develop mobility impairment as a first step in the disablement process [1]. Given the rising numbers of elderly people, there is an increasing need to identify modifiable risk factors of mobility impairment in order to prevent or delay disability onset.

In the past two decades, it has become evident that the role of vitamin D extends beyond calcium homeostasis [2]. Experimental studies have revealed vitamin D receptors (VDR) in skeletal muscle [3], [4], and vitamin D metabolites have been found to affect muscle metabolism by stimulating de novo protein synthesis, increasing the proportion of type II muscle fibers and improving muscle function [5]–[7]. Clinical studies on older people have shown that low serum levels of 25-hydroxyvitamin D (25OHD) correlate with a decrease in lower-extremity muscle strength and poorer performances in rising from a chair [8]–[11]. However, several other studies failed to show any association between vitamin D status and motor performance measures [12]–[15]. Controversial findings may result from differences in the number and type of performance tests considered, and an inadequate control over potential confounders that might impair physical performance. As an example, in several studies analyses were not stratified by gender, or they were only performed in women [10], [11], [16], introducing a significant bias since women perform less well than men, and they have lower vitamin D levels [17]. In addition, most of the studies supporting the association between vitamin D and physical performance limited their analysis to two or three tests, such as chair stands or gait tests [10], [11], [16]–[19], which are not representative of global muscle function. Another bias concerns the application of vitamin D cut-offs usually established to define the risk for osteoporosis, which are not expected to define mobility risk [10], [20]. Finally, several studies failed in controlling for confounders such as depression and cognitive impairments, two chronic conditions with a relevant impact on elderly people's performance [10], [11], [17], [19], [20]. The association between serum 25OHD concentrations and physical performance thus remains controversial and a consensus on which 25OHD levels are adequate for mobility function in older-aged people is still lacking.

The first aim of the present study was to ascertain the association between vitamin D status and mobility in a large sample of Italian older people by testing the relationship between their 25OHD serum levels and a large battery of physical performance tests, exploring balance, gait speed, coordination, upper and lower limb strength and aerobic capacity. The second aim was to identify an adequate serum 25OHD level for musculoskeletal functions in elderly men and women.

## Methods

### Data Source and Subjects

Data for this analysis are from the *Progetto Veneto Anziani* (Pro.V.A.), an observational cohort study on the Italian population aged ≥65 yrs, living in two geographical areas in the North-East of Italy (Camposampiero and Rovigo). The study population included 3099 age- and sex-stratified Caucasian participants (1245 men and 1854 women), who were randomly selected between 1995 and 1997, using a multistage stratified method. Sampling procedures and data collection methods have been described elsewhere [21]. Participants were examined at the clinics by trained physicians and nurses. Disease status was determined by integrating information from physical examination and medical records review. Disability was defined as the inability to perform 1 or more of the activities of daily living (ADLs): bathing, dressing, eating, using the toilet, or transferring. Participants who lacked serum 25OHD values (n = 272), those in wheel chairs or unable to walk (n = 89), or with leg and/or arm amputations (n = 23), and cases of hyperparathyroidism – defined as serum calcium levels >10.5 mg/dl and parathyroid hormone (PTH) levels >55 ng/L (n = 21) – were ruled out. The final sample consisted of 2694 subjects whose data on physical performance were complete. The local ethical committees of Padua University and of the Local Health Units (USSL) n. 15 and n. 18 of the Veneto Region approved the study protocol, and participants gave their written informed consent. Subjects unable to give their informed consent were not enrolled.

**Table 1:** Participants' characteristics by serum 25-hydroxyvitamin D (25OHD) quintiles in women: the PRO.V.A. Study.

	*25OHD quintiles (nmol/L)*					
	≤32 (n = 339)	>32 & ≤49 (n = 315)	>49 & ≤68 (n = 322)	>68 & ≤93 (n = 304)	>93 (n = 317)	Age-adjusted p for trend
Age (yrs)	79.9(7.5)	76.4(7.7)	75.2(6.9)	73.5(6.6)	72.4(6.0)	<.0001 (unadjusted)
BMI (kg/m^2^)	27.9(5.3)	28.1(5.1)	28.0(4.5)	28.5(4.7)	27.7(4.5)	.03
Current smokers, %	6.8	4.4	7.4	6.2	4.4	.01
Regular activity,%	49.6	62.4	67.3	77.2	80.2	<.0001
Depression,%	50.5	41.0	37.5	33.8	37.6	.07
MMSE ratio	0.72(0.23)	0.77(0.17)	0.79(0.16)	0.82(0.14)	0.83(0.13)	.006
Cardiovascular diseases, %	31.6	21.7	16.5	14.9	12.0	.003
Neuro-degenerative diseases,%	18.3	9.2	6.8	4.6	3.5	.03
Osteoarticular diseases,%	78.6	73.5	72.4	63.5	65.2	.03
Any cancer,%	7.1	8.6	6.5	6.2	7.3	.97
Visual impairments,%	36.6	26.0	23.6	20.0	18.3	.05
Diabetes,%	13.3	10.8	9.6	8.9	8.5	.10
COPD,%	7.1	7.9	3.4	4.3	2.8	.005*
***Serum levels***						
Calcium (mg/dl)	9.4(2.4)	9.4(0.4)	9.4(0.4)	9.7(5.7)	9.5(0.43)	.49
PTH (ng/L)	56.4(43.1)	44.6(20.6)	42.6(21.0)	39.4(18.9)	36.3(31.5)	<.0001
GFR (ml/min/1.73 m^2^)	64.5(19.0)	65.9(20.2)	65.9(16.9)	67.9(17.3)	68.9(16.1)	.88
Albumin (g/dL)	4.22(0.40)	4.32(0.47)	4.33(0.36)	4.36(0.31)	4.38(0.35)	.005
25OHD (nmol/L)	20.4(7.8)	41.2(4.9)	58.5(5.3)	80.1(7.2)	128.5(37.8)	

* also adjusted for smoking habit; BMI (body mass index), MMSE (Mini-Mental State Examination), COPD (Chronic Obstructive Pulmonary Diseases), PTH (parathyroid hormone), GFR (glomerular filtration rate), 25OHD (serum 25-hydroxyvitamin D).

For conversion from nmol/L to ng/ml divide by 2.496.

Numbers are mean values (and Standard Deviations) or percentage (%), as appropriate.

**Table 2:** Participants' characteristics by serum 25-hydroxyvitamin D (25OHD) quintiles in men: the Pro.V.A. Study.

	*25OHD quintiles (nmol/L)*					
	≤ 53 (n = 231)	>53 & ≤79(n = 212)	>79 & ≤103(n = 217)	>103 & ≤143(n = 219)	>143(n = 218)	Age-adjustedp for trend
						
Age (yrs)	80.6(8.0)	76.7(8.1)	75.9(7.44)	74.0(6.9)	73.5(6.4)	<.0001 (unadjusted)
BMI (kg/m^ 2^ )	26.2(4.4)	26.9(4.1)	26.7(3.7)	26.6(3.3)	27.1(3.5)	.83
Current smokers, %	24.2	20.3	20.7	26.5	22.5	.20
Regular activity, %	65	79.6	85.1	83.4	95.0	<.0001
Depression, %	35.4	22.7	20.2	19.7	17.4	.002
MMSE ratio	0.74(0.21)	0.80(0.18)	0.81(0.14)	0.84(0.12)	0.84(0.11)	.007
Cardiovascular diseases, %	38.7	28.4	28.1	23.8	22.6	.03
Neuro-degenerative diseases, %	20.4	10.3	1.8	3.6	2.3	<.0001
Osteoarticular diseases, %	49.8	49.5	46.0	37.4	36.7	.09
Any cancer,%	13.0	10.0	6.4	6.8	6.4	.02
Visual impairment,%	35.2	28.9	20.7	21.5	17.0	.1
Diabetes, %	10.4	7.6	8.8	7.8	4.6	.02
COPD,%	20.3	14.6	21.2	14.2	9.2	.13*
***Serum levels***						
Calcium (mg/dl)	9.7(2.3)	9.3(0.6)	9.4(0.4)	10.0(8.6)	9.4(0.7)	.95
PTH (ng/L)	48.3(32.8)	39.3(18.9)	39.5(18.9)	32.9(14.2)	29.4(16.5)	<.0001
GFR (ml/min/1.73 m^2^)	70.2(20.3)	72.9(19.4)	74.7(19.5)	74.3(16.9)	78.4(18.5)	.16
Albumin (g/dL)	4.23(0.43)	4.34(0.36)	4.38(0.34)	4.37(0.32)	4.42(0.32)	.0003
25OHD (nmol/L)	34.3(14)	66.1(7.7)	91.7(7.1)	121.5(11.4)	198.8(56.5)	

* also adjusted for smoking habit; BMI (body mass index), MMSE (Mini-Mental State Examination), COPD (Chronic Obstructive Pulmonary Diseases), PTH (parathyroid hormone), GFR (glomerular filtration rate), 25OHD (serum 25-hydroxyvitamin D).

For conversion from nmol/L to ng/ml divide by 2.496.

Numbers are mean values (and Standard Deviations) or percentage (%), as appropriate.

### Physical performance measures

Physical performance measures were assessed using standardized performance tests [21]:

- Tandem test (static balance ability): participants were asked to maintain balance in three different positions: a side-by-side position, a semi tandem position, and a full-tandem position. The amount of time they succeeded in remaining so, in seconds, was recorded;
- 5 timed chair stands, TCS (coordination and strength): participants were asked to stand up and sit down 5 times as quickly as possible, with their hands folded across their chest; the time taken to complete the test, in seconds, was recorded;
- Gait speed: the best performance achieved in two walks at usual pace along a 4m corridor was recorded in meter/seconds. Participants were allowed to use canes or walkers;
- 6-minute walking test, 6 mW (aerobic capacity): participants were asked to walk at their usual pace for 6 minutes, recording the distance they covered in meters [22];
- Handgrip and quadriceps strength: handgrip strength, in kg, was measured using a JAMAR hand-held dynamometer (BK-7498, Fred Sammons, Inc.). The best result obtained at two attempts with each hand was used for analyses. Knee extensor (quadriceps) and hip flexor (iliopsoas) muscle strengths were determined using a Nicholas Manual dynamometer (BK-7454, Fred Sammons, Inc.). Quadriceps strength, in Newton, was determinate in the dominant leg [23].

### Biochemical measurements

Venous blood samples were obtained after an overnight fast, centrifuged and stored at −80°C. Routine biochemical tests were performed at the city hospitals, whereas PTH and 25OHD tests were performed at the university laboratory of Padua. Serum 25OHD levels were measured by radioimmunoassay (RIA kit; DiaSorin). The intra-assay and interassay coefficients of variation for 25OHD were 8.1% and 10.2%, respectively. Serum intact PTH levels were measured using a two-site immunoradiometric assay kit (N-tact PTHSP; DiaSorin): the intra-assay and interassay coefficients of variation for PTH were 3.0% and 5.5%, respectively. Serum creatinine was measured using a standard creatinine Jaffe method (Roche Diagnostics, Germany) and glomerular filtration rate (GFR) was calculated with the MDRD formula. Serum albumin was measured using an agarose electrophoretic technique (Hydragel Protein(E) 15/30; Sebia, France).

**Table 3:** Observed physical performance measures (mean [SD]) by serum 25-hydroxyvitamin D (25OHD) quintiles in women: the Pro.V.A. Study.

	*25OHD quintiles*					
	≤32 (n = 339)	>32 & ≤49 (n = 315)	>49 & ≤68 (n = 322)	>68 & ≤93 (n = 304)	>93 (n = 317)	Age- adjusted p for trend
***Performance tests***						
**Side by side, sec**	9.9(0.6)	9.9(0.4)	9.9(0.4)	9.9(0.6)	9.9(0.1)	.68
**Semi-tandem, sec**	9.2(2.0)	9.5(1.9)	9.6(1.5)	9.7(1.4)	9.9(0.8)	.07
**Full tandem, sec**	7.9(3.0)	8.4(2.7)	8.2(2.9)	8.3(2.9)	8.5(2.8)	.30
**5 timed chair stands, sec**	16.4(10.8)	14.0(6.3)	14.0(9.1)	13.1(5.2)	12.4(3.5)	<.0001
**Gait speed, m/s**	0.55(0.20)	0.61(0.21)	0.64(0.18)	0.67(0.18)	0.70(0.18)	<.0001
**6-min walking distance, m**	221.5(117.8)	283.5(109.9)	297.3(109.1)	321.4(96.8)	332.8(86.4)	<.0001
**Handgrip strength, kg**	20.0(5.8)	22.5(5.7)	23.1(6.0)	24.1(5.9)	23.9(5.6)	<.0009
***Quadriceps strength***						
**Knee extension, N**	18.7(9.0)	19.9(7.6)	22.1(20.2)	21.4(8.2)	21.1(19.1)	.39
**Hip flexion, N**	20.9(52.0)	18.2(7.11)	20.3(9.4)	20.8(8.2)	21.1(21.9)	.89

**Table 4:** Observed physical performance measures (mean[SD]) by serum 25-hydroxyvitamin D (25OHD) in men: the Pro.V.A. Study.

	*25OHD quintiles (nmol/L)*					
	≤53 (n = 231)	>53 & ≤79 (n = 212)	>79 & ≤103 (n = 217)	>103 & ≤143 (n = 219)	>143 (n = 218)	Age-adjusted p for trend
***Performance tests***						
**Side by side, sec**	9.9(0.6)	9.9(0.7)	10(0)	10(0)	9.9(0.6)	0.43
**Semi-tandem, sec**	9.6(1.4)	9.8(0.9)	9.6(1.5)	9.8(1.1)	9.8(1.1)	0.38
**Full tandem, sec**	8.4(3.0)	9.1(2.3)	9.0(2.3)	9.0(2.3)	9.4(1.8)	0.36
**5 timed chair stands, sec**	14.5(11.8)	12.0(4.4)	12.0(4.4)	12.0(9.0)	11.0(3.2)	0.03
**Gait speed, m/s**	0.63(0.21)	0.72(0.20)	0.76(0.19)	0.77(0.17)	0.80(0.17)	<0.0001
**6-min walking distance, m**	281.6(133.8)	345.6(119.0)	349.7(119.4)	384.1(96.3)	395.8(92.5)	<0.0001
**Handgrip strength, kg**	30.5(9.1)	33.8(8.7)	34.9(8.8)	37.2(8.2)	37.1(7.9)	<0.0001
***Quadriceps strength***						
**Knee extension, N**	24.3(21.5)	30.2(30.8)	28.8(24.9)	31.0(24.2)	30.4(17.8)	0.26
**Hip flexion, N**	24.9(24.8)	31.2(31.9)	26.7(10.1)	31.2(24.1)	29.7(11.0)	0.47

**Table 5:** Adjusted estimates of physical performance mean values [mean (SE)] by serum 25-hydroxyvitamin D (25OHD) in women: the Pro.V.A Study.

*FEMALE*	*25OHD quintiles (nmol/L)*					
	≤32	>32 *&* ≤49	>49 *&* ≤68	>68 & ≤93	>93	*p* for trend
**5 timed chair stands, sec**						
*Model 1*	15.3 (0.5)	13.7 (0.4)	13.7 (0.4)	13.5 (0.4)	12.9 (0.4)	.001
*Model 2*	15.2 (0.5)	13.7 (0.4)	13.7 (0.4)	13.4 (0.4)	13.0 (0.4)	.004
**Gait speed, m/s**						
*Model 1*	0.64 (0.01)	0.64 (0.01)	0.64 (0.01)	0.65 (0.01)	0.66 (0.01)	.03
*Model 2*	0.64 (0.01)	0.64 (0.01)	0.65 (0.01)	0.66 (0.01)	0.66 (0.01)	.10
**6-minute walking distance, m**						
*Model 1*	276.1 (5.4)	300.6 (5.0)	303.2 (4.8)	309.4 (5.0)	309.7 (4.9)	<.0001
*Model 2*	280.3 (5.3)	301.8 (4.8)	304.5 (4.7)	309.9(4.8)	309.9 (4.7)	.0002
**Handgrip strength, kg**						
*Model 1*	22.3 (0.3)	23.3 (0.3)	25.5 (0.3)	23.9 (0.3)	23.0 (0.3)	.10
*Model 2*	22.5 (0.3)	23.3 (0.3)	23.6 (0.3)	24.0 (0.3)	22.9 (0.3)	.25

Notes: Adjusted mean values were obtained using the GLM procedure. Model 1 was adjusted for confounder variables (age, BMI, smoking habit, regular physical activity, season, depression, cognitive status, glomerular filtration rate (according to the MDRD formula). Model 2 was adjusted for the variables in Model 1 plus the covariates cardiovascular diseases, osteoarticular disease, COPD, and visual impairment;

SE =  standard error.

**Table 6:** Adjusted estimates of physical performance mean values [mean (SE)] by serum 25-hydroxyvitamin D (25OHD) in men: the Pro.V.A Study.

MALE	*25OHD quintiles (nmol/L)*					
	≤53	>53 & ≤79	>79 & ≤103	>103 & ≤143	>143	*p* for trend
**5 timed chair stands, sec**						
*Model 1*	13.1 (0.5)	11.4 (0.5)	12.1 (0.4)	11.4 (0.4)	11.6 (0.4)	.05
*Model 2*	13.0 (0.5)	11.4 (0.5)	12.0 (0.4)	11.5 (0.4)	11.6 (0.4)	.08
**Gait speed, m/s**						
*Model 1*	0.71 (0.01)	0.76 (0.01)	0.78 (0.01)	0.77 (0.01)	0.77 (0.01)	.0001
*Model 2*	0.72 (0.01)	0.76 (0.01)	0.78 (0.01)	0.77 (0.01)	0.77 (0.01)	.0006
**6-minute walking distance, m**						
*Model 1*	330.3 (7.6)	362.1 (7.2)	361.9 (6.8)	379.0 (6.7)	378.6 (6.6)	<.0001
*Model 2*	334.6 (7.4)	362.6 (6.9)	366.0 (6.6)	376.4 (6.5)	376.6 (6.3)	<.0001
**Handgrip strength, kg**						
*Model 1*	34.7 (0.6)	34.8 (0.5)	35.1 (0.5)	36.6 (0.5)	35.9 (0.5)	.01
*Model 2*	34.8 (0.6)	34.8 (0.5)	35.3 (0.5)	36.5 (0.5)	35.9 (0.5)	.03

Notes: Adjusted mean values were obtained using the GLM procedure. Model 1 was adjusted for confounder variables (age, BMI, smoking habit, regular physical activity, season, depression, cognitive status, glomerular filtration rate (according to the MDRD formula). Model 2 was adjusted for the variables in Model 1 plus the covariates cardiovascular diseases, osteoarticular disease, COPD, and visual impairment;

SE =  standard error.

### Statistical analyses

Participants' characteristics were summarized using means (± standard deviations) for continuous variables and counts and percentages for categorical variables. Given the gender-related differences, all data analyses were stratified by sex. Means and proportions were calculated for sex-specific quintiles of the distribution of 25OHD serum levels. For continuous variables normal distributions were tested using the Shapiro Wilk test. Differences in categorical variables were examined using the C*hi*-square test. Age-adjusted *p* values for trends were calculated, checking the differences between means of covariates by quintiles of vitamin D status using analysis of variance (ANOVA). General linear models (GLM) were used to examine the independent association between 25OHD levels and performance tests. The association between performance and vitamin D status was tested considering 25OHD levels both as a continuous variable (per unit of 25OHD) and as a categorical variable (sex-specific quintiles). The presence of a nonlinear (quadratic) effect of 25OHD concentrations was examined but did not emerge, so only the linear associations were modeled. Known factors associated with 25OHD levels and/or physical functionality were examined for inclusion in the analyses and two multivariate models were obtained. Age, smoking habit (never/former vs current smoker), body mass index (BMI; calculated as the weight in kg/height in meters squared), physical activity (defined as ≥4 h/week in the previous month of at least moderate physical activity, e.g. brisk, walking, cycling, swimming, dancing, gardening or physical exercising), cognitive impairment (Indexed Mini-Mental State Examination score <0.8; [24]), depression (defined as a score ≥11 on the Geriatric Depression Scale [25]), season of the year (November–February vs March–October) and GFR were added as confounders in the first model (Model 1). Among adjudicated diagnoses of cardiovascular diseases (CVD: coronary heart disease, congestive heart failure, cerebrovascular disease, peripheral artery disease, hypertension), diabetes, chronic obstructive pulmonary disease (COPD), osteoarthritis (including hand/knee/hip osteoarthrosis, hip fracture), neurodegenerative diseases (Parkinson, dementia), cancer, and visual impairments, those with a p-value <.10 at bivariate analysis (i.e. CVD, osteoarthritis, COPD and visual impairment) were considered as covariates and added into Model 2, including both confounders and covariates. Also variables that could act as intermediate factors of altered 25OHD levels- i.e. PTH concentrations and osteoporosis- were added to Model 2. To identify the best 25OHD levels for musculoskeletal functions in the elderly men and women, we conducted a loess analysis, where loess smoother is a form of locally weighted regression line using a weighted average of a set of data points at each part of the curve and is robust to outlying values. The procedure, applied to the whole data set in both genders, included the same variables as the multivariate models.

**Figure 1:**
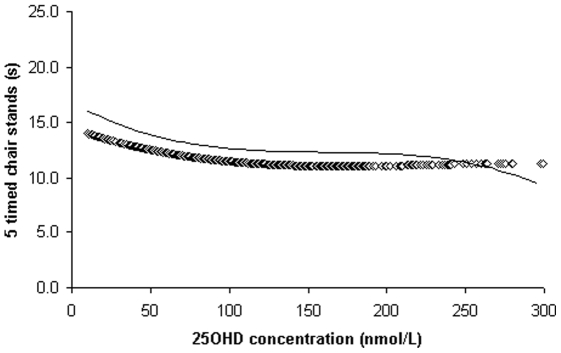
Loess regression plots of 5 timed chair stands measure (seconds) by 25-hydroxyvitamin D (25OHD) concentrations; straight line–for women and diamond line ◊◊ for men.

**Figure 2:**
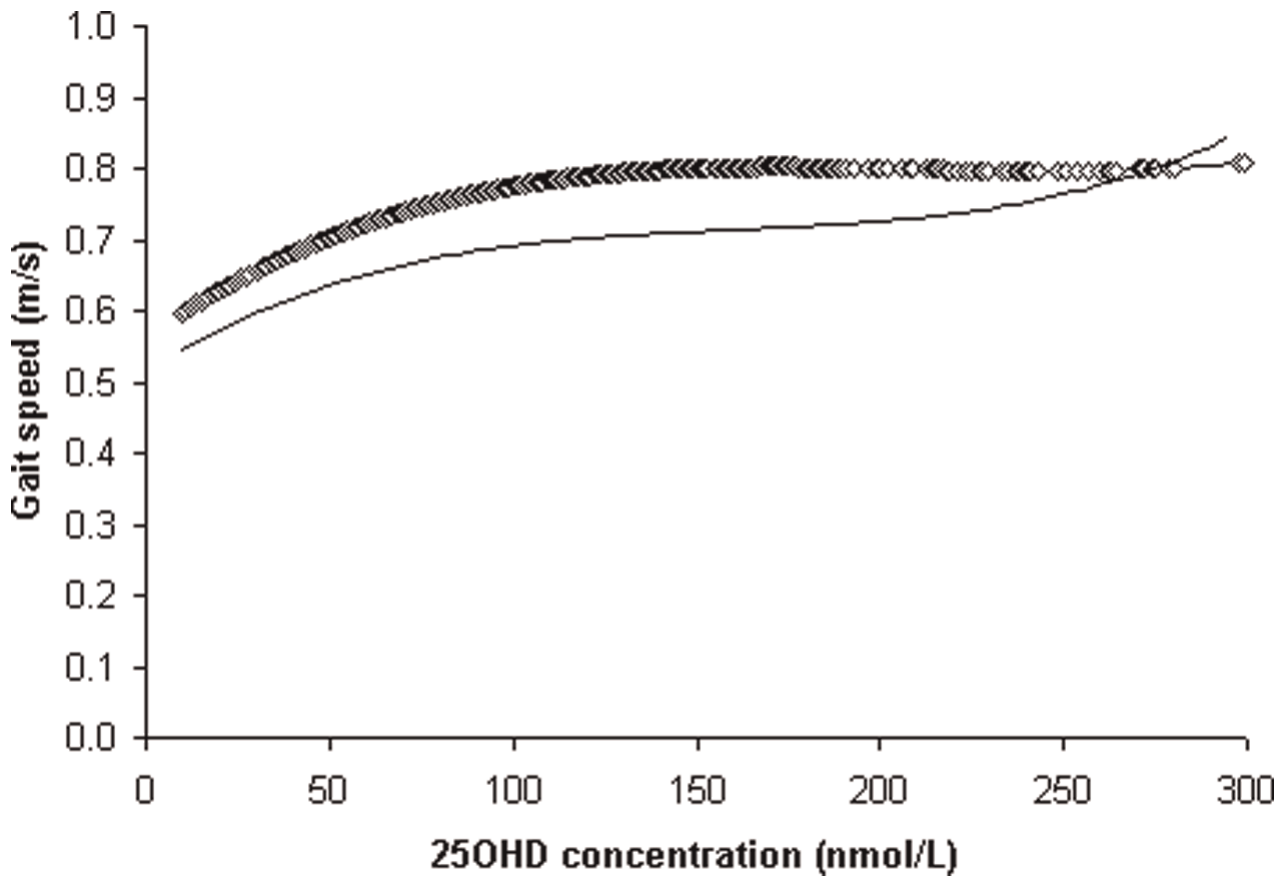
Loess regression plots gait speed (meter/seconds) by 25-hydroxyvitamin D (25OHD) concentrations; straight line–for women and diamond line ◊◊ for men.

**Figure 3:**
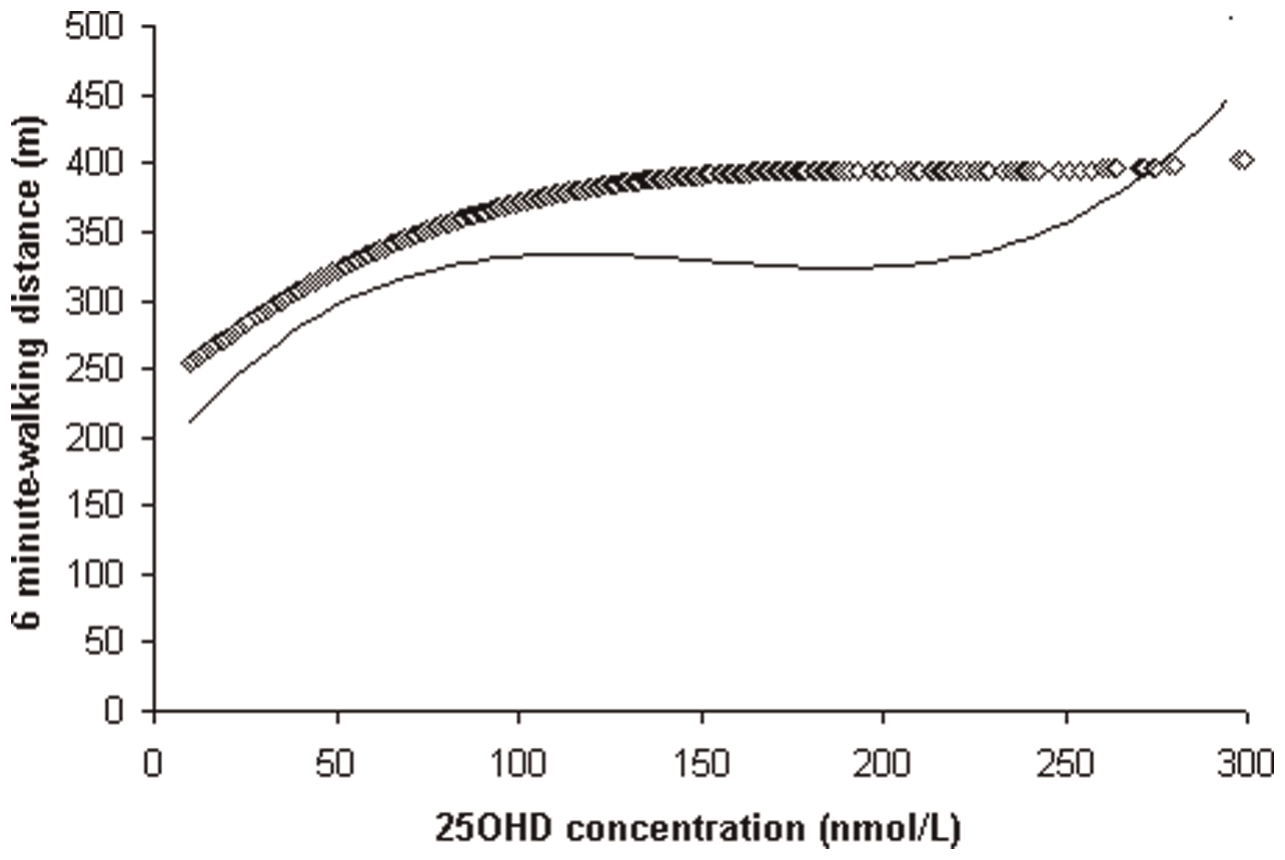
Loess regression plots of 6-minute walking distance (meters) by 25-hydroxyvitamin D (25OHD) concentrations; straight line–for women and diamond line ◊◊ for men.

**Figure 4:**
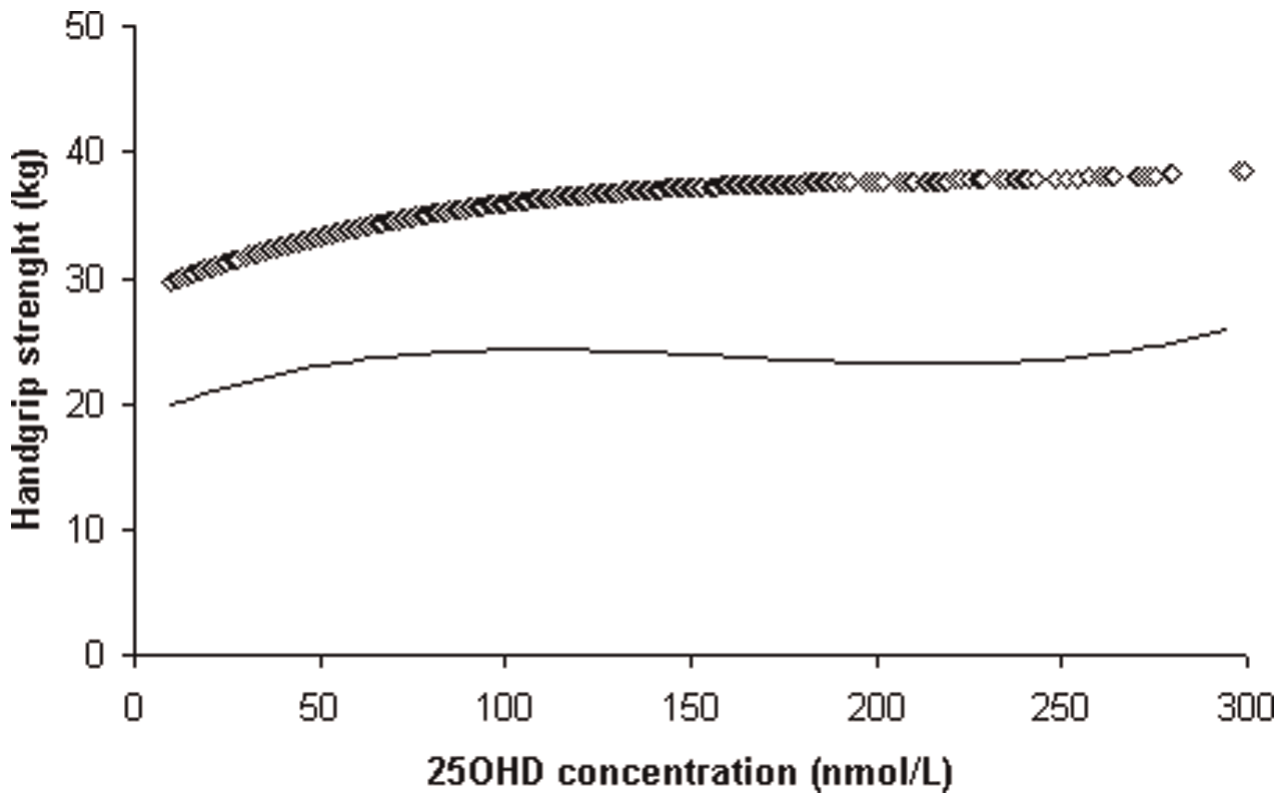
Loess regression plots of handgrip strength (kg) by 25-hydroxyvitamin D (25OHD) concentrations; straight line–for women and diamond line ◊◊ for men.

All analyses were performed using the SAS rel. 9.13 (Cary NC: SAS institute). All statistical tests were two-tailed and statistical significance was assumed for a p-value <.05.

## Results

### Participants' characteristics

The sample consisted of 2694 community-dwelling elderly subjects (1597 F and 1097 M). They were not currently disabled in ADL_s_. Their mean age (±SD) was 75.6 y (±7.5; range 65–98 y) in women, and 76.2 y (±7.8; range 65–99) in men. The mean serum 25OHD level was 65.0 nmol/L (±41.3; range: 2.5–329) in women, and 101.9 nmol/L (±62.4; range: 2.5–441) in men. Vitamin D deficiency, often defined as a serum 25OHD level <50 nmol/L [26], was present approximately in the 40% of the women and in the 20% of the men; whereas severe deficiency (25OHD <25 nmol/L; [26]) was identified in 13.5% of women and 5.9% of men.

The age-adjusted characteristics of the participants, classified by the quintiles of their 25OHD levels, are shown in Tables 1 and 2, respectively for women and men. Participants in the lowest 25OHD quintile were significantly older than those with higher levels of 25OHD (*p* for trend <.0001). After adjusting for age, both male and female participants in the lowest quintiles were significantly less active, more depressed and more cognitively impaired than participants in the highest quintiles.

### Association between 25OHD levels and performance measure

The age-adjusted mean physical performance measures by 25OHD quintiles are shown for women and men in Tables 3 and 4, respectively. No differences in 25OHD quintiles emerged for tandem test performance and quadriceps strength in either gender, so these tests were excluded from further analyses. Significant linear associations were found for TCS test (*p for trend* <.0001 in women;.03 in men), gait speed and 6 mW test (*p for trend* <.0001, in both genders), and for handgrip strength (*p for trend* <.0009 in women; <.0001 in men). The multivariate adjusted mean measures obtained for these performance tests are shown in Tables 5 and 6, for women and men respectively. After controlling for confounders and covariates, a significant linear trend was still evident for the 6 mW test in both genders (p = .0002 in women; <.0001 in men), for the TCS test in women (p = .004), and for gait speed (p = .0006) and handgrip strength (p = .03) in men. Further adjustment for PTH levels and osteoporosis slightly attenuated the associations between 25OHD levels and physical performance measures (details not shown).

When the associations between physical performance and vitamin D levels were examined using serum 25OHD levels as a continuous variable, a significant linear association emerged in the adjusted model for both genders only for the 6 mW test: the *β* coefficient [*SE*] per unit of serum 25OHD was 0.15 [0.05] in women, p = .007; and 0.14 [0.05] in men, p = .004. Further adjustment for PTH levels and osteoporosis did not affect the associations between 25OHD levels and this performance test.

### Loess analyses for 25OHD levels and performance measures

The association between 25OHD concentrations and performance measures is shown for TCS, gait speed, 6 mW distance and handgrip strength tests in Figures 1, 2, 3 and 4 respectively. The time taken to complete the TCS test decreased significantly with increasing levels of 25OHD in women only and most of the improvements occurred between 20 to 100 nmol/L of 25OHD. In men but not in women, gait speed increased significantly for 25OHD levels up to 100 nmol/L, most of the improvement occurring at concentrations ranging from 50 to approximately 75 nmol/L. Handgrip strength improved in men with increasing level of vitamin D up to 100 nmol/L. The 6 mW distance continued to increase up to 25OHD serum levels of 100 nmol/L in both genders. No further significant improvements in these four motor performances were seen for 25OHD levels >100 nmol/L, in both genders.

## Discussion

The association between vitamin D and performance tests exploring mobility impairments has become a clinically hot topic in the last two decades.

In our large, population-based sample of community-dwelling Italian elderly subjects, we found a significant positive association between 25OHD concentrations and 4 of the 6 performance tests habitually used to assess mobility impairment. The association was strong for TCS, gait speed, 6 mW distance and handgrip strength. After controlling for several confounders, 25OHD levels were clearly associated with TCS results in women, but not in men, and with gait speed and handgrip strength only in men.

Such gender-related differences are probably explained by the significantly higher 25OHD levels seen in men, who were less likely to have vitamin D deficiency than women. This large sex-difference in 25OHD levels between genders is not so unusual, as reported in previous studies [17]. In the Longitudinal Aging Study Amsterdam (LASA), longer times to complete the TCS were only observed in participants with 25OHD <50 nmol/L [18]. In our study, less than 20% of the men had 25OHD levels lower than 50 nmol/L, and this might explain why an association with TCS in this cohort was only seen in women.

A slower walking speed coinciding with 25OHD levels <50 nmol/L has already been reported too [10], [11], [18]. In our study, gait speed increased with serum 25OHD levels in both genders in the unadjusted models, but after controlling for confounders, this association persisted in men but not in women. Unfortunately, we cannot compare our results with those of other cross-sectional studies because gender-specific analyses are often unavailable and gait speed is not always considered as a measure in its own right, but scored as part of the Short Physical Performance Battery [11], [18], [20].

The Rancho Bernard Study found vitamin D status associated with handgrip strength in elderly men, but not in older women [17], whereas in Zamboni et al [27], 25OHD <40 nmol/ml correlated with arm strength in women, but not in men. Differences vis-à-vis other studies might be explained by the larger number of potential confounders for which our analyses were adjusted. Another, more likely explanation for all the sex-related differences found in our study is that men might have more preserved muscle strength than women of the same age, therefore women are already below strength and speed thresholds at which vitamin D might have a significant impact. Other potential mechanisms, such as differences in vitamin D receptor gene polymorphisms, might also be behind the sex-specific differences identified [28].

Despite the above mentioned differences in performance and serum 25OH levels, the “pattern” of the association between vitamin D concentrations and performance measures is similar in both genders, so that the lower the 25OHD level, the lower the observed performance.

Among all the performance measures considered, the 6 mW distance was the motor test most strongly related to vitamin D status in both genders. Results in the 6 mW test, regarded as a measure of aerobic capacity, have recently been correlated with cardiovascular risk [19], [29], while the relationship between vitamin D and the cardiovascular system is still unclear. Ours is the first study to be performed on a large sample of elderly individuals, confirming the linear association between 25OHD levels and the 6 mW distance in both genders, even after adjusting for numerous confounders. Serum 25OHD concentrations might therefore be able to predict the aerobic capacity of elderly subjects: the higher the vitamin levels, the higher the tolerance of exercise.

In our study, higher vitamin D serum levels are clearly associated with higher prevalence of regular physical activity and lower frequency of disease. In this sample of elderly subjects one of the most practiced physical activities was gardening (details not shown). We might suppose that this out-door activity might be related to higher sun-exposition, leading to higher serum levels of 25OHD. On the other hand, regular physical activity was stated by the healthiest elderly subjects, with the lowest prevalence of comorbidity. Given that the cross-sectional design of this study does not allow the knowledge of sequential events, vitamin D levels might be cautiously interpreted as a biomarker of good health status and good quality of life.

Our study did not confirm the association between vitamin D and static balance tests or quadriceps strength [16], [30], [31]. In a few cross-sectional studies, an impaired static balance was only found in women with serum 25OHD levels <25 nmol/L [10], [18], [32], while several other studies enrolling elderly subjects with higher vitamin D levels failed to demonstrate any association between 25OHD concentrations and the tandem test. Interventional studies also failed to show any significant improvement in static balance after vitamin D supplementation [16]. Similar discrepancies have been found for quadriceps strength, with which several studies failed to show any association with 25OHD levels [13]–[16]. The results from EPIDOS study confirmed that leg extensor strength was associated with age, sex and BMI, but not with 25OHD or PTH concentrations [13]. Given the above-mentioned published reports, our results confirm the lack of any association between vitamin D levels and quadriceps strength, highlighting the complexity of this poorly understood relationship.

In our study, loess analyses confirmed that 25OHD levels as close as 100 nmol/l are clearly associated in elderly people with a faster walking time, better performances in rising from a chair, higher upper limb strength, and greater aerobic capacity. Thresholds for adequate vitamin D have already been defined, but they are generally based on PTH levels, not on physical performance outcomes. One study drawn from the NHANES III data concluded that it was desirable to reach 25OHD concentrations of at least 40 nmol/L for optimal lower extremity function [11]. Some have suggested that older adults should be supplemented to maintain 25OHD levels at 70 nmol/L at least [18]. Therefore defining adequate 25OHD thresholds for both muscle skeletal and extra skeletal outcomes is more than a challenge. In our study, the identified vitamin D threshold for physical performance outcome was slightly higher than those previously reported and in contrast with Bischoff-Ferrari *et al* [11], we found no significant decline in performance among subjects with the highest of 25OHD levels, going against any hypothesis that higher vitamin D concentrations might be toxic to motor function. However for upper levels of serum 25OHD sparse data are available, particularly regarding long-term effects of chronically high concentrations, thus a margin of safety for public health recommendation is prudent. According to the last report of the Institute of Medicine on the tolerable upper vitamin D levels, serum 25OHD concentration above 125 nmol/L should raise concerns among clinicians about potential adverse effect, particularly on extra skeletal outcomes [33].

The present study has limitations. A participation bias probably attenuated the results, since the participants were probably the healthiest. This might explained why in our population-based sample vitamin D levels were significantly higher than those reported in previous studies on elderly Italian subjects [34], [35]. Differences in methods used to measure 25OHD make it difficult to compare optimal levels with those observed in other studies [36], although methodological differences would not affect the linear association seen between 25OHD and physical function. Moreover the cross-sectional design of our study did not allow us to formulate hypothesis on the causality of the relationship between vitamin D and performance.

The main strengths of our study lie in its population-based design and large sample size, comprising a proportion of men and women representative of the general older population in northern Italy. A further strength relates to the large number of confounders and adjudicated diseases investigated, and the numerous performance tests conducted to explore different dimensions of mobility.

In conclusion, serum concentrations of 25OHD close to 100 nmol/L seem to be associated to greater benefit for musculoskeletal functions in our elderly community-dwelling subjects. Given the high prevalence of vitamin D insufficiency in the elderly population of northern Italy, ageing people should be given supplementation to keep their 25OHD levels as nearest as possible to this threshold, in order to preserve their physical performance. Besides additional researches are needed for consensus on 25OHD threshold in order to avoid problems of both under and overtreatment.
